# Comparative *In Vitro* Effects of Calcineurin Inhibitors on Functional Vascular Relaxations of Both Rat Thoracic and Abdominal Aorta

**DOI:** 10.1155/2013/718313

**Published:** 2013-06-18

**Authors:** Ashok Jadhav, Venkat Gopalakrishnan, Ahmed Shoker

**Affiliations:** ^1^Department of Pharmacology, College of Medicine, University of Saskatchewan, Saskatoon, SK, Canada S7N 5E5; ^2^Department of Medicine, College of Medicine, University of Saskatchewan, Saskatoon, SK, Canada S7J 5B6; ^3^Saskatchewan Transplant Program, Royal University Hospital, 103 Hospital Drive, Saskatoon, SK, Canada S7N 0W8

## Abstract

*Background and Aim*. Calcineurin inhibitors (CNIs) have shown to develop hypertension in transplant patients. The *in vitro* incubation effects of cyclosporine (CsA) and tacrolimus (Tac) on vascular relaxations of rat thoracic aorta (TA) and abdominal aorta (AA) need to be investigated. *Methods*. The optimal concentrations of CsA (1.0 mg/mL) and Tac (0.1 mg/mL) used to compare endothelium-dependent (acetylcholine (ACh)) and endothelium-independent (sodium nitroprusside (SNP)) vascular relaxation against the agonists in phenylephrine (PE-) constricted TA and AA of 13-week-old male Sprague Dawley rats (*n* = 6). *Results*. In TA, the maximal vasodilator response elicited by ACh (control: *I*_max_ 98%) was significantly (*P* < 0.01) inhibited by CsA (*I*_max_ 10%) but not by Tac (*I*_max_ 97%). In AA, (control: IC_50_ 50 nM; *I*_max_ 100%) CsA (IC_50_ 7 **μ**M; (*P* < 0.01) showed strong sensitivity to inhibit ACh-dependent vascular relaxation than Tac (IC_50_ 215 nM (*P* < 0.05); *I*_max_ 98%). CsA and Tac failed to affect the inhibitory responses to SNP in both TA and AA. *Conclusion*. CsA exerts profound inhibitory effect on endothelium-dependent vasodilatation as compared to Tac in both TA and AA. Aortic rings from the thoracic region are more sensitive to CNIs, since the vasodilator response to ACh is solely mediated by NO while in the AA, ACh likely recruits other endothelial mediators besides NO to maintain vasodilatation.

## 1. Introduction

The post-transplantation vasculopathy is an important complication after organ transplantation [1]. The initial pathological event is thought to be an allograft endothelial dysfunction, a condition in which there is decreased generation of endothelium-derived relaxing factor, nitric oxide (NO). Either its synthesis is reduced or its increased quenching by oxidative stress is thought to result in increased vascular smooth muscle (VSM) tone and elevation in blood pressure that is accompanied by endothelial damage and loss of homeostatic regulatory property of the vascular wall [2]. Diminished NO function is thought to play a major role in the progression of post-transplantation vasculopathy [3]. Transplantation immunosuppressants such as calcineurin inhibitors (CNIs) have substantial effects on vascular reactivity, especially on NO synthesis [3]. The main two CNI drugs namely, cyclosporine A (CsA) and tacrolimus (Tac), have been suggested to impair vascular endothelial function via decreased generation of NO release along with increased generation vasoconstrictor peptide, endothelin-1 (ET-1) that leads to elevated vascular tone and resultant hypertension [4–8]. It is now well accepted that these effects of CsA and Tac are due to their direct effects on the vasculature that are independent of their effects on the kidney [9]. In contrast, one study has proposed that administration of CsA promotes enhanced vasoconstriction of rat thoracic aortic (TA) rings via its direct effects on VSM cells, and it may not affect endothelium-dependent, NO-mediated vasodilatation [10]. The same group of investigators have also demonstrated that prolonged *in vivo* administration of CsA leads to both endothelium-dependent (vasodilator response to acetylcholine (Ach)) as well as endothelium-independent (sodium nitroprusside (SNP)) reduction in vasodilator responses in thoracic but not in the abdominal portions of rat aortic rings [11]. However, a rationale for such a remarkable degree of regional differences between segments of rat aortic rings was not adequately explained in that study. The study failed to show whether *in vitro* incubation with a CNI such as CsA or Tac would affect the vascular responses to agonists. The results with the CNIs on the regulation of vascular function are indeed very conflicting owing to differences in study protocol, drug doses and treatment durations [4–11]. To the best of our knowledge, there has not been any information on the relative impact of a comparative effect of both CsA and Tac on the responses to vasodilator agonists on TA versus abdominal aorta (AA). 

In an attempt to address the above, the major goal of the present study is to examine whether acute *in vitro* incubation for a limited and short time of exposure for a 30 min duration with optimal and clinically relevant concentrations of either CsA (1 mg/mL) or Tac (0.1 mg/mL) would affect endothelium-mediated vasodilatation evoked by ACh and/or endothelium-independent vasodilatation induced by a direct VSM relaxing agonist such as SNP in phenylephrine (PE)-constricted rat aortic rings. This is the first report on the comparison of the differential and acute effects of *in vitro* addition of both CsA and Tac on endothelium-intact TA versus AA of rat aortic rings. 

## 2. Methods

### 2.1. Animals

The studies were performed using 13-week-old male Sprague-Dawley rats (300 to 350 g) purchased from Charles River Laboratories (St. Constant, Quebec, Canada). The experimental protocol was approved by the Animal Ethics Board at the University of Saskatchewan conformed to the Guide for the Care and Use of Laboratory Animals stipulated by the Canadian Council on Animal Care and the National Institutes of Health publication.

### 2.2. Materials

Acetylcholine chloride, phenylephrine hydrochloride, sodium nitroprusside, and all the salts used in the preparation of Krebs buffer were of analytical grade obtained from Sigma-Aldrich Canada Ltd. (Oakville, Ontario, Canada). Cyclosporine A (Novartis, Sandimmune^®^ Injection i.v.) was obtained from Novartis Pharmaceuticals, Canada Inc. (Dorval, Quebec, Canada), while tacrolimus (Prograf, i.v.) was purchased from Astella Pharma Canada Inc. (Markham, Ontario, Canada). Isoflurane and heparin sodium were obtained from Abbott Laboratories Limited and Sandoz, Canada Inc., respectively (Montreal, Quebec, Canada).

### 2.3. *In Vitro* Study with Rat Thoracic and Abdominal Aortic Rings

*In vitro* studies were performed using ring preparations of TA and AA of rat aorta. The vessels were quickly isolated from male Sprague-Dawley rats after the animals were anaesthetised with isoflurane. The effects of CsA and Tac induced changes in tension responses of isolated rat TA and AA were determined by suspending these preparations in organ baths containing 10 mL Krebs' buffer (in mM: 120, NaCl; 4.8, KCl; 1.2, MgCl_2_; 1.8, CaCl_2_; 1.2, KH_2_PO_4_; 25, NaHCO_3_; 11, glucose; pH 7.4 gassed with 95% O_2_, 5% CO_2_ at 37°C) maintained under a resting preload tension of 2 gr. as described previously [12, 13]. Adequate care was taken to insert the hooks without damaging the endothelium. The aortic rings were first contracted with a submaximal concentration (~EC_80_ level) of *α*_1_ selective agonist, PE (1 *μ*M). Once the steady state tonic response was attained, the tissues were washed in normal Krebs buffer for a period of 1 hr, and the response to the same concentration of PE was repeated to ascertain that the sustained tonic response to PE is reproduced. Then, a fixed concentration of ACh (10 *μ*M) was added to assess the extent of vasodilatation in PE-constricted rings. If vasodilatation to ACh was >90%, it was considered as an endothelium-intact preparation since ACh evokes vasodilatation only in vessels with intact endothelium. Once a steady tonic response was reached following the addition of PE, cumulatively increasing concentrations of either ACh (1 nM–10 *μ*M) or SNP (1 nM–10 *μ*M) were added in such a way that the next concentration was added only after the response to the previous concentration had plateaued. The data collected were considered as control (before treatment) and compared with data obtained after treatment of drugs in the same tissue since PE-evoked responses could be reproduced in the same preparation as long as the inhibitory drug is exposed only once. Tissues were washed 3 times and allowed to recover by repeated washing for a minimum of 1 hr. The same tissues were incubated with either CsA (1 mg/mL) or Tac (0.1 mg/mL) in Krebs buffer for 30 min. Then, the tissues were constricted with PE (1 *μ*M) to reach steady state tonic response, and cumulatively increasing concentrations of either ACh or SNP were repeated in the presence of either CsA or Tac until the maximal inhibition (*I*_max⁡_) of steady state tonic vasoconstrictor response to PE could be reached. Thus, the changes in isometric tension evoked by ACh and SNP in the presence or absence of both CsA and Tac in PE-constricted rings were determined in the vessels of the same rat (*n* = 6). The tension responses were recorded in gram on a chart programme (Chart V5.0.1) using a Powerlab/8SP data acquisition system (AD Instruments Pvt. Ltd., Sydney, Australia).

### 2.4. Statistical Analysis

The inhibition of tension evoked following the additions of each concentration of ACh or SNP in PE constricted vessels were normalized as the percentage inhibition of steady state tonic response evoked by PE. Since the inhibitory effect reached closer to 100%, the concentration of either ACh or SNP required to produce 50% of the maximal inhibition (IC_50_) as well as the percentage of maximal inhibition (*I*_max⁡_) values for each condition of incubation could be generated from each concentration-inhibition response curve (CRC) using Prism software (Graph Pad Inc., La Jolla, CA). The data obtained using vessels isolated from one rat for a specific condition of incubation were pooled, and the mean value from that rat was considered as *n* = 1. Then, similar experiments were replicated with blood vessels isolated from 6 rats (*n* = 6). Thus, the final mean ± SEM values shown in the results section represent the data gathered from several rats. The differences in mean ± SEM values between different conditions of incubation were analysed using one-way ANOVA, followed by Tukey post hoc test, and the data were considered significant when the *P* value was <0.05. However, for assigning the level of significance, the closest *P* value reached was provided in Section 3.

## 3. Results

Addition of either the vehicle (peanut oil 10%) in which higher concentrations of both CsA or Tac were prepared or the addition of appropriate concentrations of either CsA (1.0 mg/mL) or Tac (0.1 mg/mL) maintained in the organ bath for a period of 30 min in Krebs buffer failed to affect the basal tone, while PE-evoked steady tonic responses of TA or AA portions of rat aortic rings were enhanced and comparable (data not shown).

**Figure 1:**
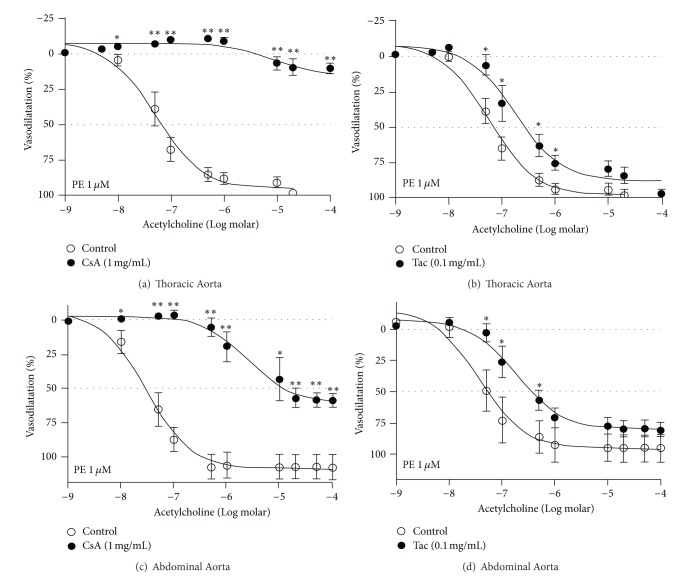
The panel compares the concentration-inhibition response curves (CRC) to acetylcholine (ACh) determined in phenylephrine (PE—1 *μ*M)-constricted thoracic ((a) and (b) panels) and abdominal ((c) and (d) panels) aortic rings with intact endothelium either in the presence or absence of cyclosporine (CsA—1 mg/mL) or tacrolimus (Tac—0.1 mg/mL) maintained in the incubation medium for a period of 30 min after isolation from 13-week-old male Sprague Dawley rats (*n* = 6). *P < 0.05 and **P < 0.01 compared to respective data in the control group.

Addition of ACh led to concentration dependent inhibition of PE-evoked steady state tonic response between 1 nM and 1 *μ*M in both TA and AA. The CRC determined from several experiments is shown (Figures 1(a)–1(d)). There were no significant differences in either IC_50_ or *I*_max⁡_ control values for ACh determined in the absence of either CsA or Tac in both rat TA and AA (Figure 1 and Table 1). Addition of CsA (1.0 mg/mL) led to almost complete inhibition of responses to ACh in TA with the *I*_max⁡_ value being significantly much lower (10% ± 2% *P* < 0.001) than the control value for ACh (98% ± 1%). The acute inhibitory effect of CsA was so profound in endothelium-intact TA rings, and this was similar to the abolition of the inhibitory effect of ACh seen in endothelium-denuded vessels (Figure 1(a)). The addition of Tac also inhibited the responses to ACh but the inhibitory effects were comparatively much lower as there was only a right-ward shift in the CRC to Ach; the IC_50_ value for ACh was right shifted from a control value of 68 ± 12 nM to 222 ± 60 nM in the presence of Tac. While the degree of right shift was significant (*P* < 0.05), Tac incubation failed to affect the maximal inhibitory effect of ACh as the *I*_max⁡_ value was not different between control versus Tac treatment groups (Figure 1(b), Table 1). While a similar pattern of inhibitory effect of ACh was also seen in rat AA, the effects of both CsA and Tac were relatively less sensitive in rat AA in comparison to the profound inhibitory effects exerted by CsA and Tac in TA (Figures 1(c) and 1(d)). Addition of CsA shifted the CRC from a mean ± SEM IC_50_ control value of 50 ± 18 nM to 7 ± 3 *μ*M (*P* < 0.001). The *I*_max⁡_ value was also reduced from complete inhibition (100% ± 2%) to a level of 58% ± 5%, and the inhibitory effect was significant (*P* < 0.01; Table 1). In comparison to CsA, Tac evoked inhibition of ACh vasodilatation was less sensitive (*P* < 0.05) with right-ward shift in the CRC to ACh (IC_50_ value increased from a control value 50 ± 18 nM to 215 ± 59 nM by Tac), and the *I*_max⁡_ was reduced from 100% ± 2% to 80% ± 6% (*P* < 0.05, see Figures 1(c) and 1(d) and Table 1).

**Figure 2:**
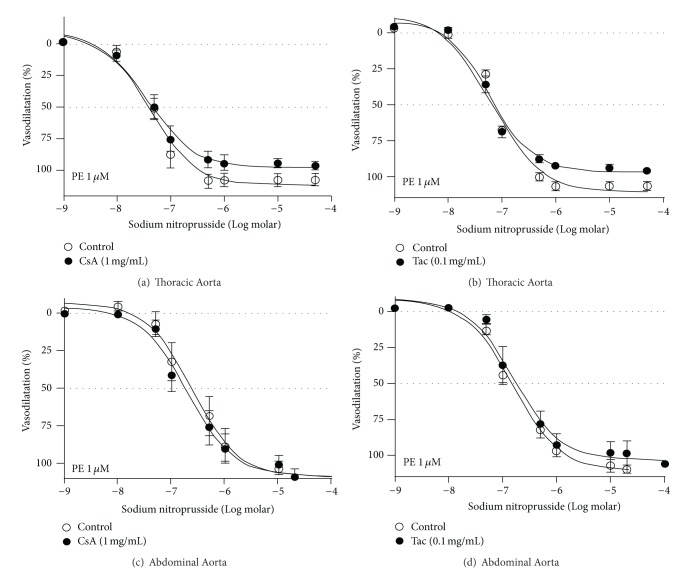
The panel compares the CRC to sodium nitroprusside (SNP) determined in PE (1 *μ*M)-constricted thoracic ((a) and (b) panels) and abdominal ((c) and (d) panels) aortic rings in the presence or absence of cyclosporine (CsA—1 mg/mL) and tacrolimus (Tac—0.1 mg/mL). Each data point is mean ± SEM value obtained from aortic rings isolated from 6 rats.

The addition of SNP also produced concentration-dependent vasodilatation with complete inhibition of responses in PE-constricted TA and AA rings before the addition of either CsA or Tac (control data shown in Figure 2 and Table 2). While the *I*_max⁡_ values reached 100% in either preparations, TA rings were relatively more sensitive with lower IC_50_ values to SNP than AA rings. Addition of either CsA or Tac failed to alter significantly the IC_50_ and *I*_max⁡_ values for SNP in either TA or AA of rat aortic rings despite their sensitivity differences (Figures 2(a)–2(d) and Table 2).

## 4. Discussion

The major finding reported in the present study is the observation that CsA inhibits endothelium-dependent vasodilatation to a much greater extent than Tac, and this effect is more pronounced in thoracic than in abdominal region of the rat aorta. The acute *in vitro* comparative data reported in the present study is consistent with the incidence of hypertension development in previously normotensive liver or heart transplant recipients treated with CsA and Tac. Hypertension developed in about 80%–90% of patients those are receiving CsA compared to a relatively lower (50%–60%) incidence of hypertension in patients receiving Tac treatment [14–16]. Another recent report has also shown that Tac showed a significantly lower score (*P* < 0.05) than CsA when augmentation of arterial stiffening and small and large artery compliance index were considered following CsA and Tac treatment in renal transplant recipients receiving these two agents [17]. The significance of our present functional *in vitro* study is discussed in the light of the above clinical observations. 

Both CsA and Tac are well-established CNIs that act as immunosuppressive agents by inhibiting calcineurin, a calcium, and calmodulin-dependent serine-threonine protein phosphatase 3 in T lymphocytes [18]. Elevation in cytosolic free calcium level in T lymphocytes following antigen binding to its T-cell receptors leads to activation of calcineurin which promotes the transcription of several cytokine genes leading to organ rejection. CNIs provide protection against transcription of these cytokines activated by calcineurin. Although both agents inhibit calcineurin, they target different cytoplasmic-binding proteins. CsA inhibits cyclophilin A while Tac targets FK-binding proteins to inhibit calcineurin [19]. Such subtle differences in their mechanism and site of action could contribute to their differential effects in their vascular effects. The protective immunosuppressive effect of CNIs also comes with the cost of adverse effects of hypertension [16]. A number of different mechanisms have been proposed for the development of hypertension such as (i) impaired endothelium-dependent vasodilatation due to decreased generation of NO from the endothelium, (ii) increased production of vasoconstrictor peptide, ET-1, from the endothelium, (iii) endothelium-independent enhanced vasoconstriction at the level of vascular smooth muscle, (iv) increased renal vasoconstriction, (v) elevated sympathetic drive followed by activation of renin-angiotensin system leading to oxidative stress and the accompanying vasoconstriction, and (vi) defects in renal sodium handling resulting in sodium retention [17]. Although all of these mechanisms could contribute to elevation in blood pressure, earlier studies have proposed that CNIs exert direct vascular effects apart from its effects on the kidney [17]. Earlier studies with both CNIs have proposed that they could regulate endothelium-dependent as well as endothelium-independent vasodilatation [4–11]. Thus, it is important to establish the direct vascular effects of these agents by performing *in vitro* studies using isolated VSM preparations. The possible mechanism by which the CNIs such as CsA and Tac could alter the phosphorylation states of serine-threonine residues from the endothelial No synthase (eNOS) leading to reduced expression and activity of eNOS. Thus, decrease in NO generation in the endothelial cells has been documented previously [7, 8]. Similarly, inhibition of endothelium-independent dilatation evoked by direct nitro-vasodilators such as SNP has also been suggested [10, 11]. However, there are no studies that compare the effects of both CsA and Tac in any particular vasculature. The present study has addressed this key issue. Although this paper is not the first to report differences in physiological variable between the CsA and Tac, but comparative in vitro functional study was performed to characterize the differential effect on both endothelium-dependent and -independent vascular relaxation in rat TA and AA.

It is now well established that Tac is 10 to 100 times more potent than CsA as a CNI [20]. Thus, the concentration that we chose for comparison in the determination of the acute *in vitro* effects of CsA (1 mg/mL) and Tac (0.1 mg/mL) is appropriate and justified. We demonstrate that addition of such optimal low concentration of CsA for 30 min led to abolition of endothelium-dependent vasodilator responses to ACh in thoracic rat aortic rings whereas incubation with Tac for the same duration led to only partial inhibition of the responses. Moreover, Tac was much less potent as an inhibitor of ACh responses in the abdominal portion of the aortic ring. A regional difference in vasculotoxic effect of CsA with profound inhibition of endothelium-dependent (ACh-mediated) and marginal endothelium-independent (SNP-induced) vasodilatation in thoracic but not in abdominal region of aortic rings has been previously demonstrated [11]. In this study, aortic rings were harvested for *in vitro* examination of tension responses following chronic oral administration of CsA to normotensive Sprague-Dawley rats at a dose of either 20 mg/kg for a prolonged period of either ten days or administration of a dose of 50 mg/kg for 7 days. After this treatment, the tissues were harvested, and *in vitro* responses were determined. In contrast, in our *in vitro* study we harvested both TA and AA from normal SD rats and mounted in organ bath; later we have taken vascular dilator response before and after exposure of CNIs for 30 min with TA and AA rings. Our data show that the inhibitory effect of CsA was more profound in TA as compared to AA. Verbeke et al. could not provide a convincing explanation as to why CsA selectively attenuated the endothelium-dependent ACh mediated vasodilatation in TA but not in AA of rat [11]. Moreover, there was no inhibition of SNP-induced vasodilatation in our study, and this confirms that the low concentration of the CNIs we employed did not affect the cGMP mediated vasodilator responses to a directly acting nitro-vasodilator such as SNP at the level of VSM cells. We have also ascertained that incubation with either CsA or Tac enhanced the contractile response to PE (*α*_1_ adrenoceptor agonist) in either TA or AA; however, response pattern was similar between TA and AA. Others have also shown that there are no segmental differences in vasoconstrictor responses evoked by PE between thoracic and abdominal rat aortic rings since PE activates one subtype of *α*_1D_ adrenergic receptors in either regions [21]. Thus, our data confirm that the *in vitro* effects of CNIs are linked to inhibition of endothelium-dependent vasodilatation especially after incubation for 30 min in organ bath.

It is now established that ACh-induced endothelium-dependent vasodilatation is mediated solely by the release of NO in major conduit vessels such as aorta [22, 23]. As the vessel diameter decreases, ACh could recruit, besides NO, other endothelial mediators such as endothelium-derived hyperpolarization factor (EDHF) and/or prostacyclin (PGI_2_) to maintain vasodilatation even if the NO release is blunted [23, 24]. Thus, in smaller blood vessels such as the main mesenteric artery and its branches (mesenteric arterioles) or the descending abdominal aorta, the vasodilator responses to ACh persist even if the NOS inhibitor, L-NAME is included in the medium as endothelium-dependent vasodilatation to ACh is maintained by generation of EDHF [25–28]. CNIs exert selective blockade of eNOS expression and its activity by regulating the serine threonine phosphorylation sites on NOS [8]. Thus, CNIs selectively inhibit only eNOS and do not affect Ca^2+^ dependent EDHF activation that is linked to small and intermediate conductance Ca^2+^activated K^+^channels (SK_Ca_ and IK_Ca_) in the descending portion of the rat aorta. NO mediates the vasodilator response to ACh in TA. CsA and Tac inhibit NO-mediated vasodilatation to ACh due to serine-threonine phosphorylation of eNOS. It is likely that the effect of CsA is profound in reducing eNOS activity while Tac is relatively less sensitive in the thoracic region following its acute CNI incubation. This is consistent with previous finding that CsA affect vascular function [29, 30]. There is no back-up or compensatory EDHF mediated vasodilatation. Therefore, there is abolition of ACh-mediated vasodilatation in the presence of CsA and a significant level of inhibition of vasodilatation to ACh following the addition of Tac. While in the AA, the compensatory EDHF-mediated vasodilatation maintains the responses to ACh despite eNOS blockade exerted by CNIs in this region. Our functional data suggests that CsA is more potent in blocking eNOS than Tac in both TA and AA rings with the order of inhibitor potency being CsA > Tac and the order of sensitivity for the inhibition of responses being TA > AA. This is also consistent with what is seen in clinical settings with the incidence of hypertension being relatively high following CsA compared to Tac treatment although the overall adverse effects with regard to nephrotoxicity and neuotoxicity incidences following Tac was relatively higher [18, 31]. Previous finding with *in vitro* incubation of Tac with mouse aorta in organ bath showed impaired endothelial-dependent relaxation response to ACh which was linked to altered intracellular calcium release affecting eNOS [32]. Clearly, more studies are warranted with investigation of the responses to ACh determined in the combined presence and absence of eNOS inhibitor (L-NAME) and EDHF inhibitors (apamin + TRAM-34) along with CsA and Tac maintained in the incubation medium to resolve these issues.

## 5. Conclusions

Our short term *in vitro* functional study reveals that CsA is more potent than Tac in the inhibition of endothelium-dependent, ACh-induced vasodilatation in both TA and AA. There are regional variations in the responses to CNIs with TA being more sensitive to blockade than AA. In addition, we also found that CNIs did not affect the SNP-induced vasodilatation in thoracic and abdominal aorta.

## Figures and Tables

**Table 1:** The comparative effects of *in vitro* incubation with two calcineurin inhibitors (CNI), cyclosporine (CsA, 1.0 mg/mL), or tacrolimus (Tac, 0.1 mg/mL) for 30 min duration on acetylcholine (ACh)-induced vasodilator responses in phenylephrine (PE, 1 *µ*M)-constricted thoracic and abdominal rat aortic rings with intact endothelium.

Region	Incubation condition	ACh-DRC: Mean ± SEM values	
		IC_50_	*I*_max⁡_
Thoracic aorta	Control	68 ± 16.4 nM	98% ± 1.3%
	CsA (1.0 mg/mL)	9.5 ± 2.55 *µ*M**	10% ± 2.2%**
	Tac (0.1 mg/mL)	222 ± 60.2 nM*	97% ± 2.7%

Abdominal aorta	Control	50 ± 17.8 nM	100% ± 0.0%
	CsA (1.0 mg/mL)	7.0 ± 3.14 *µ*M**	58% ± 4.94%**
	Tac (0.1 mg/mL)	215 ± 58.6 nM*	80% ± 5.79%*

*P < 0.05 and **P < 0.01 compared to respective control. Each value is mean ± SEM of several experiments performed using rat aortic rings isolated from 6 rats for each condition of incubation.

Note: vehicle control studies with castor oil alone yielded closely similar values like control data performed in the presence of with Kreb's buffer alone.

**Table 2:** The comparative effects of *in vitro* incubation with either CsA (1 mg/mL) or Tac (0.1 mg/mL) for 30 min. Duration on sodium nitroprusside (SNP-) evoked vasodilator responses in phenylephrine (PE-1 *µ*M)-constricted rat aortic rings with intact endothelium.

Region	Incubation condition	SNP-DRC: Mean ± SEM values	
		IC_50_	*I*_max⁡_
Thoracic aorta	Control	75 ± 4.0 nM	104% ± 4.4%
	CsA (1.0 mg/mL)	50 ± 19.0 nM	96% ± 3.0%
	Tac (0.1 mg/mL)	58 ± 7.0 nM	96% ± 1.5%

Abdominal aorta	Control	158 ± 24.2 nM	110% ± 2.9%
	CsA (1.0 mg/mL)	342 ± 179.1 nM	104% ± 5.3%
	Tac (0.1 mg/mL)	194 ± 52.4 nM	105% ± 1.79%

Each value shown above is mean ± SEM of several experiments performed using aortic rings isolated from a minimum of 6 rats for each condition of incubation.

## Abbreviations

ACh: Acetylcholine
CNI: Calcineurin inhibitors
CRC: Concentration-response curve
CsA: Cyclosporine A
eNOS: Endothelial nitric oxide synthase
ET-1: Endothelin-1
EDHF: Endothelium-derived hyperpolarization factor
NO: Nitric oxide
PE: Phenylephrine
Tac: Tacrolimus
SNP: Sodium nitroprusside
VSM: Vascular smooth muscle
TA: Thoracic aorta
AA: Abdominal aorta.
